# The Phenomenon of Hematocephalus: A Comprehensive Review of the Literature

**DOI:** 10.31662/jmaj.2023-0039

**Published:** 2023-04-07

**Authors:** Atsushi Saito

**Affiliations:** 1 Department of Neurosurgery, Hirosaki University School of Medicine Graduate School of Medicine, Hirosaki, Japan

**Keywords:** hematocephalus, hydrocephalus, intraventricular hemorrhage

Hematocephalus is a noncommunicating hydrocephalus caused by intraventricular hematoma, as described by Martin et al ^(1)^. Intraventricular hematoma includes massive clots spreading in all four ventricles and partial clots obstructing cerebrospinal fluid circulation in the foramen of Monro and aqueduct and foramen of Magendie. The authors have described in detail the characteristics of hematocephalus; however, its basic mechanism and clinical management have not been fully discussed, as indicated by the authors. I suppose that many neurosurgeons agree with the authors’ opinion that simple intraventricular drainage is not always successful in the management of hematocephalus associated with massive intraventricular hematoma. Difficulties in management are as follows: the bleeding point is often unknown based on pre-operative imaging; emergent cases cannot allow conventional angiographical examination; even large drainage catheters can sometimes clog with clots of hematoma; intracatheter administration of fibrinolytic agents can cause rebleeding; the selection of neuroendoscopies is not simple; flexible- or rigid-type surgical devices for flexible neuroendoscopy for the removal of hematoma have not been fully developed; and hematocephalus includes variable causes of hemorrhage, which has complex pathological states. Moreover, clinical states often need emergent management, which can be challenging. The paper is of considerable interest to clinicians. The definition of hematocephalus is based on the report by Benes et al ^(2)^. The recent categorization of hematocephalus can provide new perspectives on pathological states and therapeutic strategies. The present paper is believed to contribute to the accurate categorization of the knowledge on hydrocephalus caused by intraventricular hematoma and development of new approaches to clinical management.

## Article Information

### Conflicts of Interest

None
